# Minimally invasive segmentectomy and lobectomy for peripheral stage IA1–2 non-small-cell lung cancer: a case-matched cohort study from a UK Centre

**DOI:** 10.1093/icvts/ivad204

**Published:** 2023-12-13

**Authors:** Alessandro Brunelli, Amr Rushwan, Demetrios Stefanou, Polivious Drosos, Nilanjan Chaudhuri, Richard Milton, Peter Tcherveniakov, Kostas Papagiannopoulos, Laura Valuckiene

**Affiliations:** University of Leeds, School of Medicine, Leeds, UK; Department of Thoracic Surgery, St James’s University Hospital, Leeds, UK; Department of Thoracic Surgery, St James’s University Hospital, Leeds, UK; Department of Thoracic Surgery, St James’s University Hospital, Leeds, UK; Department of Thoracic Surgery, St James’s University Hospital, Leeds, UK; Department of Thoracic Surgery, St James’s University Hospital, Leeds, UK; Department of Thoracic Surgery, St James’s University Hospital, Leeds, UK; Department of Thoracic Surgery, St James’s University Hospital, Leeds, UK; Department of Thoracic Surgery, St James’s University Hospital, Leeds, UK; Department of Thoracic Surgery, St James’s University Hospital, Leeds, UK

**Keywords:** Segmentectomy, Lung cancer, Lobectomy, Survival, Recurrence, Video-assisted thoracoscopic surgery, Robotic surgery

## Abstract

**OBJECTIVES:**

The objective of this study is to compare in a real-world series the short- and long-term results of segmentectomy and lobectomy for peripheral clinical stage IA non-small-cell lung cancer (NSCLC).

**METHODS:**

Single-centre cohort study including a series of consecutive patients undergoing minimally invasive segmentectomy or lobectomy for peripheral (outer third of the lung) clinical stage IA NSCLC (January 2017–August 2022). Propensity score case matching analysis generated 2 matched groups of patients undergoing segmentectomy or lobectomy. Short-term (morbidity and mortality) and long-term [overall survival and event-free survival (EFS)] outcomes were compared between the 2 matched groups. EFS was calculated by including death resulting from any cause and any recurrence as events.

**RESULTS:**

Propensity score generated 118 pairs of patients undergoing minimally invasive segmentectomy or lobectomy. The median follow-up was 30 months (95% confidence limits (CL) 4–64). The median postoperative length of stay was 4 days in both groups. Ninety-day mortality was similar (segmentectomy 2.5% versus lobectomy 1.7%, *P* = 1). Three-year overall survival [segmentectomy 87% (76–93) versus lobectomy 81% (72–88), *P* = 0.73] and EFS [segmentectomy 82% (72–90) versus lobectomy 78% (68–84), *P* = 0.52] did not differ between the groups. Loco-regional recurrence rate [segmentectomy 4.2% (5/118) versus lobectomy 9.3% (11/118), *P* = 0.19] was similar despite a lower nodal upstaging [segmentectomy 3.4% (4/118) versus lobectomy 14% (17/118), *P* = 0.005]. The occurrence of compromised resection margins (pR1 or pR uncertain) was similar between the groups [segmentectomy 7.6% (9/118) versus lobectomy 9.3% (11/118), *P* = 0.81].

**CONCLUSIONS:**

This observational series confirms the non-inferiority of segmentectomy compared to lobectomy in treating peripherally located stage IA NSCLC.

**Figure:**
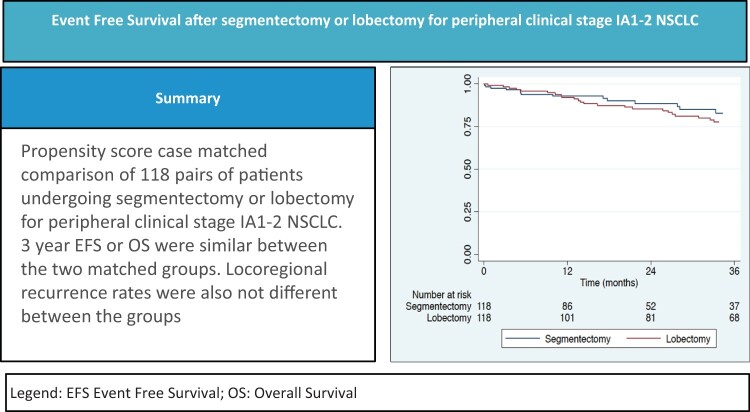

## INTRODUCTION

Recent randomized trials have shown that segmentectomy is at least non-inferior to lobectomy for peripheral clinical stage IA1–2 non-small-cell lung cancer (NSCLC) patients [1, 2]. Patients receiving a sublobar resection had similar disease-free survival (DFS) and overall survival (OS) compared to those undergoing lobectomy. The 2 trials have however shown a large difference in the magnitude of reported outcomes, with the North American study finding a 12–16% poorer OS and DFS in both treatment groups. This difference may be in part explained by differences in patient and tumour-related characteristics in the 2 studies.

Compared to the JCOG trial, the CALGB trial found more than twice as higher rate of loco-regional recurrence, which was in both studies 3%–4% more frequent in segmentectomy patients.

Albeit reassuring the above-mentioned nuances warrant to be checked in the local clinical practice. In particular, there is a paucity of studies from Europe compared to the overwhelming majority published from Asian and North American centres. With this rationale, we aimed to compare the short- and long-term outcomes of patients undergoing segmentectomy or lobectomy in our unit for peripheral cT1a–bN0 NSCLC.

## PATIENTS AND METHODS

### Ethics statement

The study was reviewed by the Research and Innovation Department of Leeds Teaching Hospitals and classified as service evaluation not requiring formal review by the research ethics committee and individual consent for this retrospective analysis was waived.

This is a retrospective cohort analysis of patients undergoing lobectomy or segmentectomy at the Department of Thoracic Surgery, St James’s University Hospital, Leeds from January 2017 to August 2022. Our unit is a tertiary referral centre. The study was written according to the STROBE (Strengthening the reporting of observational studies in epidemiology) checklist (www.strobe-statement.org). All consecutive patients submitted to minimally invasive (video-assisted thoracoscopic or robotic) anatomic segmentectomy or lobectomy for clinical stage IA1–2 (tumour smaller than 2 cm) and with the tumour located peripherally in the outer third of the lung were included in the analysis. Clinical staging was based on the 8th edition of the tumour–node–metastasis staging system. Only patients with a proven NSCLC at definitive pathology were included in the analysis.

For both lobectomy and segmentectomy, cases initiated minimally invasively and converted to open surgery were included in the analysis as per intention to treat.

The extent of the procedure (lobectomy or segmentectomy) was at the discretion of the surgeon. Segmentectomies were in general reserved for patients deemed at increased risk for lobectomy (compromise segmentectomies), or as intentional procedure when the solid component of the tumour was smaller than 2 cm and no nodal disease was evident at preoperative staging (clinical stage IA). Most of the segmentectomies were performed by 2 surgeons who had more experience with this procedure. It is important to note that the study period preceded the publication of the randomized trials and some of the surgeons were still reluctant to offer a sublobar resection to fit patients. All operations were performed under the care of 6 board-certified general thoracic surgeons. As most of the patients included in this study were operated during a period before the publication of the 2 phase 3 trials [1, 2] most of the surgeons in our unit still preferred to offer a lobectomy as a surgical option in fit patients. The choice of the procedure reflected therefore the preference of the individual surgeon and their surgical expertise in this procedure.

All patients were discussed at a multidisciplinary team meeting where the indication for surgery was agreed.

All patients were clinically staged using positron emission tomography (PET)–computed tomography (CT) and endobronchial ultrasound (EBUS)-esophageal ultrasound (EUS) nodal sampling in case of suspected hilar or mediastinal enlarged or PET-avid lymph nodes. Patients with positive lymph nodes detected preoperatively through EBUS were considered unsuitable for sublobar resections.

Segmentectomies were performed by the individual dissection and division of the segmental arteries, bronchus and veins. Lymph node frozen section was only performed in case of a highly suspicious nodal disease. A systematic lymph node dissection was performed in all patients including lymph nodes at the foot of the segmental hilum. The intersegmental planes were identified using the inflation-deflation technique and following the anatomic structures of the segment. They were divided using mechanical stapler devices.

All patients were extubated in the operating room and monitored for few hours in a recovery unit before being transferred to a specialized thoracic ward. Perioperative management was in accordance with our enhanced recovery program [3] with early mobilization and feeding, postoperative physical rehabilitation under the supervision of specialized physiotherapists, opioid-sparing pain control and digital chest drainage management. Cardiopulmonary complications occurring within 30 days from surgery or while the patient still hospitalized were recorded and included the followings: respiratory failure requiring reintubation or a mechanical ventilation for longer than 24 h, pneumonia, atelectasis requiring bronchoscopy, pulmonary embolism, atrial arrhythmia requiring pharmacological or electrical cardioversion, acute myocardial ischaemia, acute cardiac failure, stroke and acute renal failure. Complications were defined according to the joint STS-ESTS definitions [4].

### Statistical analysis

Follow-up information (including date and cause of death) was obtained by data retrieved from the centralized electronic clinical information system of the hospital which records date of death and any medical treatment or access to care including occurred in other regional Hospitals. All patients were followed up through March 2022. No patient was lost at follow-up. OS was measured from the date of surgery to the date of death from any cause and censored at the date of last follow-up for survivors. Event-free survival (EFS) was measured from the date of surgery to the date of death from any cause or lung cancer recurrence, whichever occurred first, and censored at the date of last follow-up for survivors without recurrence.

Loco-regional recurrence was defined as recurrent disease in the lung, the hilar nodes or mediastinal nodes. All other recurrence was deemed to be systemic.

Patients undergoing lobectomy and segmentectomy were compared. To adjust for possible confounders, a propensity case-matched analysis was performed. Propensity score was calculated using logistic regression analyses without replacement using a calliper including the following variables: age, gender, body mass index, forced expiratory volume in 1 s expressed as percentage of predicted values, carbon monoxide lung diffusion capacity expressed as percentage of predicted value, presence of coronary artery disease, cerebrovascular disease, diabetes, chronic kidney disease, performance score, radiologic size of the tumour, PET SUVmax value and consolidation-to-tumour ratio. A 1:1 nearest neighbour matching without replacement was applied, using a calliper of 0.20 of the pooled standard deviation of the logit estimate [5]. Standardized difference (%) was used to assess adequacy of balance between the groups (>0.2 being inadequately balanced) [6].

In addition, a Cox proportional hazard regression analysis was performed including all patients with a peripheral tumour 2 cm or smaller in size. The analysis was repeated for OS and EFS using as independent co-variates age, gender, body mass index, forced expiratory volume in 1 s expressed as percentage of predicted values, carbon monoxide lung diffusion capacity expressed as percentage of predicted value, presence of coronary artery disease, cerebrovascular disease, diabetes, chronic kidney disease, performance score, radiologic size of the tumour, PET SUVmax value and consolidation-to-tumour ratio.

There were no missing data in this series, including follow-up information.

Survival end points were characterized with the use of the Kaplan–Meier estimator. A stratified log-rank test was used to estimate the *P*-value for testing differences between the groups, with radiologic tumour size and PET SUVmax value as stratification factors. Hazard ratios and their confidence intervals were estimated with the use of Cox proportional-hazards models with robust indicators. Survival analysis was reported at 3-year follow-up. To compare survival up to 3 years, we truncated follow-up at this time point.

In a *post hoc* analysis, we explored the treatment effect on lung cancer-related death as compared with other causes of death, with a competing regression analysis with robust indicators, in which deaths from causes other than lung cancer were considered a competing event. Cumulative incidence functions were estimated with the use of the Gray method [7] and the associated hazard ratios and confidence intervals were estimated by means of the Fine–Gray hazard model [8].

Postoperative outcomes (cardiopulmonary morbidity, mortality, recurrence rates, nodal upstaging rates) between matched groups were compared using the Wilcoxon matched-pairs signed rank test for numeric variables and McNemar’s test for categorical variables.

All tests were performed using the Stata 15.0 statistical software (Stata Corp, College Station, TX, USA).

## RESULTS

Three hundred eighty-six patients received a minimally invasive lobectomy (248) or segmentectomy (138) for a confirmed or presumed NSCLC located in the outer third of the lung and staged as cIA1–2 (cT1a or cT1b and cN0). NSCLC was confirmed by definitive pathology after 118 segmentectomies and 236 lobectomies. Eighty-seven segmentectomies were performed by one of the surgeons, whilst 17 were performed by a second surgeon. The remaining 17 segmentectomies included in this analysis were performed by the other 4 surgeons. Lobectomies were more evenly distributed among the surgeons.

These patients represent 87% and 77% of all segmentectomies and lobectomies performed in our Institution during the same period for clinical stage IA1–2 NSCLC.

Tables 1 and 2 show the baseline characteristics before and after matching of the patients undergoing segmentectomy and lobectomy.

**Table 1:** characteristics of patients undergoing lobectomy or segmentectomy for peripheral clinical stage IA1–2 before matching

Variables	Lobectomy (no. 236)	Segmentectomy (no. 118)	SD
Age	69.0 (8.0)	69.9 (7.8)	0.12
Gender male	102 (43%)	44 (37%)	0.12
FEV1%	89.8 (20.1)	92.0 (20.8)	0.10
DLCO%	74.9 (17.4)	76.1 (19.0)	0.06
BMI (kg/m^2^)	26.9 (5.1)	26.9 (4.9)	0.01
CAD	23 (9.7%)	14 (12%)	0.07
CVD	13 (5.5%)	6 (5.1%)	0.02
Diabetes	29 (12%)	8 (6.8%)	0.19
Radiologic size (mm)	17.3 (5.7)	15.4 (6.9)	0.29
Solid tumour (C/T ratio = 1)	141 (60%)	37 (31%)	0.59
PET SUVmax > 2.5	180 (76%)	64 (54%)	0.47

Results are expressed as means and standard deviation for numeric variables and count and percentages for categoric variables.

BMI: body mass index; CAD: coronary artery disease; CVD: cerebrovascular disease; DLCO: carbon monoxide lung diffusion capacity; FEV1: forced expiratory volume in 1 s; SD: standardized difference; C/T ratio: consolidation to tumour ratio; PET SUV: positron emission tomography standardised uptake value.

**Table 2:** characteristics of patients undergoing lobectomy or segmentectomy for peripheral clinical stage IA1–2 after matching

Variables	Lobectomy (no. 118)	Segmentectomy (no. 118)	SD
Age	68.3 (8.6)	69.9 (7.8)	0.19
Gender male	54 (46%)	44 (37%)	0.17
FEV1%	90.7 (20.7)	92.0 (20.8)	0.06
DLCO%	76.2 (19.4)	76.1 (19.0)	0.01
BMI (kg/m^2^)	27.3 (5.2)	26.9 (4.9)	0.08
CAD	11 (9.3%)	14 (12%)	0.08
CVD	8 (6.8%)	6 (5.1%)	0.07
Diabetes	17 (14%)	8 (6.8%)	0.25
Radiologic size (mm)	19.5 (6.2)	15.4 (7.0)	0.63
Solid tumour (C/T ratio = 1)	73 (62%)	37 (31%)	0.64
PET SUVmax > 2.5	102 (86%)	64 (54%)	0.75

Results are expressed as means and standard deviation for numeric variables and count and percentages for categoric variables.

BMI: body mass index; CAD: coronary artery disease; CVD: cerebrovascular disease; DLCO: carbon monoxide lung diffusion capacity; FEV1: forced expiratory volume in 1 s; SD: standardized difference; C/T: consolidation to tumour ratio; PET SUV: positron emission tomography standardised uptake value.

Propensity score case matching yielded 2 groups of 118 pairs. Despite matching the 2 groups were still unbalanced in terms of more solid and hypermetabolic tumours in the lobectomy patients. Patients undergoing lobectomy had slightly larger tumours.

The conversion rate was 4.2% (5 patients) in the segmentectomy group and 6.8% (8 patients) in the lobectomy group (*P* = 0.41).

The median length of stay was 4 days in both groups (75% CI 3–6).

Cardiopulmonary morbidity rate was 14% in both groups (16 patients in each group) (*P* = 1).

Ninety-day mortality rate was similar between the 2 groups (3 patients after segmentectomy 2.5% and 5 patients after lobectomy 4.2%, *P* = 0.72).

### Survival

**Figure 1:**
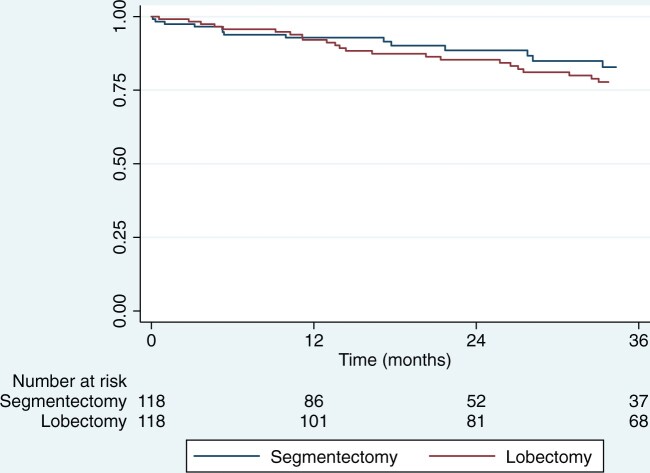
Kaplan–Meier graphs of event-free survival in matched groups of patients after segmentectomy or lobectomy for peripheral clinical stage IA1–2 non-small-cell lung cancer.

The median follow-up was 35 months (IQR 16–53). All patients analysed in this study were followed up until the end of March 2022 (follow-up index = 1). In the matched population, lobectomy was similar to segmentectomy for EFS (hazard ratio for disease recurrence or death, 1.26; 95% confidence interval, 0.69–2.33). Three-year EFS was 79% (95% CI 68–87) after segmentectomy and 76% (95% CI 68–83) after lobectomy (stratified log-rank test, *P* = 0.28) (Fig. 1).

Similarly, no difference was observed between matched groups for OS (hazard ratio for death after lobectomy, 1.13; 95% confidence interval 0.55–2.36).

**Figure 2:**
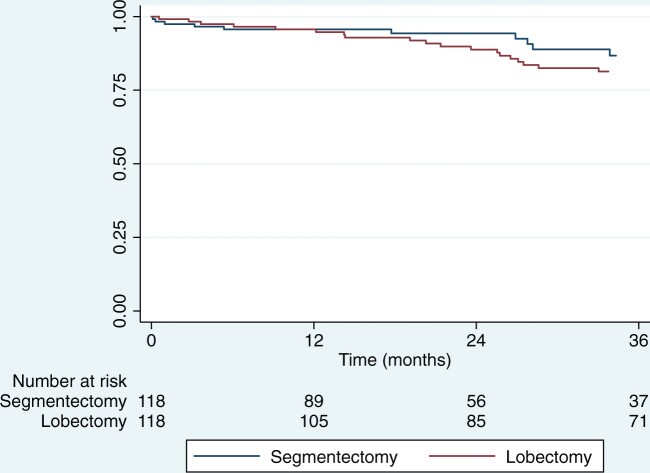
Kaplan–Meier graphs of overall survival in matched groups of patients after segmentectomy or lobectomy for peripheral clinical stage IA1–2 non-small-cell lung cancer.

Three-year OS was 85% (95% CI 75–91) after segmentectomy and 82% (95% CI 73–88) after lobectomy (stratified log-rank test, *P* = 0.73) (Fig. 2).

Competing regression analysis with deaths from causes other than lung cancer considered a competing event showed that the subhazard ratio for lung cancer-specific death was 2.2 after lobectomy (95% CI 0.57–9.0, *P* = 0.25).

**Figure 3:**
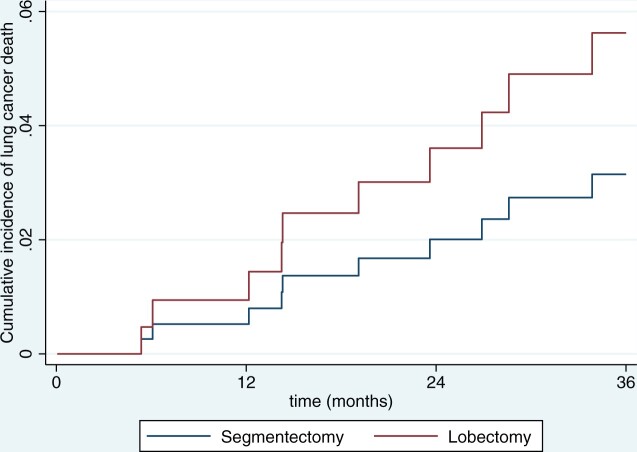
Cumulative incidence of cancer-specific death after segmentectomy or lobectomy for peripheral clinical stage IA1–2 non-small-cell lung cancer.

Figure 3 shows the cumulative incidence of lung cancer-related deaths after lobectomy and segmentectomy.

The incidence of loco-regional recurrence was 4.2% after segmentectomy (5 patients) and 9.2% (11 patients) after lobectomy. Competing regression analysis with deaths from causes other than lung cancer considered a competing event showed that the subhazard ratio for loco-regional recurrences was 2.3 after lobectomy (95% CI 0.8–6.4, *P* = 0.11).

Nodal upstaging occurred in 4 patients after segmentectomy (3.4%) and 16 patients after lobectomy (13%), *P* < 0.001.

The median distance of the tumour from the resection margin in pR0 cases was 15 mm after segmentectomy (75% CL 5–24) and 30 mm after lobectomy (75% CL 15–40, *P* < 0.0001).

Compromised margins (pR1 or pR uncertain) occurred in 9 patients after segmentectomy (7.5%) and in 13 patients after lobectomy (11%) (*P* < 0.001).

The incidence of loco-regional recurrence in this subset of patients was similar between the 2 operations (33% after segmentectomy and 18% after lobectomy, *P* = 0.62).

We also performed Cox’s proportional hazard regression analyses including all patients (unmatched analysis) with a peripheral tumour of 2 cm or smaller. After adjusting the analysis for patient and tumour-related variables, the extent of operation was not significantly associated with OS (HR for lobectomy 0.66, 95% CI 0.31–1.40, *P* = 0.28) nor for EFS (HR for lobectomy 0.72, 95% CI 0.39–1.34, *P* = 0.30). When the analysis was limited to tumour with a consolidation-to-tumour ratio of >0.5, the extent of the operation remained not significantly associated with OS (HR for lobectomy 0.70, 95% CI 0.31–1.62, *P* = 0.41) nor with EFS (HR for lobectomy 0.81, 95% CI 0.37–1.80, *P* = 0.60).

## DISCUSSION

### Background and rationale

The recent publication of 2 phase 3 trials [1, 2] showing the non-inferiority of sublobar resection compared to lobectomy for peripheral tumour smaller than 2 cm in fit patients sparked the interest for this type of procedure. Real-world scenario, however, may be different from those set for controlled randomized trials. The case mix of patients may vary and the limit between intentional and compromise segmentectomies may be less defined. In addition, a large group of patients may receive segmentectomies for situations in which this procedure appears particularly convenient, such in cases of undetermined nodules in which a sublobar resection may offer both diagnosis and cure, or in patients with multiple lesions, in whom sparing lung parenchyma may increase the chances of radical treatment. In some countries in Europe lung cancer screening is being implemented and a widespread adoption will likely occur in the entire continent in the near future. This will lead to a surge in the detection of early-stage lung cancer amenable for segmentectomy.

It is important in our view to test the oncological safety of segmentectomy shown in the RCTs in a real-world setting. For this reason, we analysed our patients undergoing segmentectomy or lobectomy for peripheral clinical stage IA1–2 for NSCLC.

### Main findings

After a median follow-up of 35 months, we were not able to find any difference in EFS or OS between matched pairs of patients with peripheral tumours smaller than 2 cm operated on by segmentectomy or lobectomy.

The 3-year EFS and OS were similar to those extrapolated from the Kaplan–Meier curves reported in the CALGB trial, although the survival rates at this follow-up time were not formally reported in that study [1].

We found a similar incidence of loco-regional recurrences after segmentectomy compared to lobectomy. This incidence was considerably lower compared to the CALGB trial (4.2% in our study vs 13% in the CALGB trial) [1]. The incidence of loco-regional relapse after lobectomy in our study was similar to the one found in the CALGB trial in this group (12% in our study vs 10% in the CALGB trial).

The loco-regional recurrence rate found in the segmentectomy group was also lower than the one reported in the JCOG trial (4.2% in our study vs 11% in the JACOG study). On the other hand, we found a higher loco-regional recurrence rate in our lobectomy group (12% in our study vs 5% in the JCOG trial) [2]. The lower incidence of loco-regional recurrences in our study compared to the 2 randomized trials may partly be explained by a shorter follow-up in our study (median 2 years in our segmentectomy group vs 7 years in the 2 randomized trials). Another notable difference of our stay compared to the 2 randomized trials was the fact that we included patients with all tumours morphologies, from pure ground glass opacity to solid ones whilst in the JCOG and CALGB studies only patients with a consolidation-to-tumour ratio of >0.5 were included. When only this subgroup of patients was analysed in the current study (52% of segmentectomies), we were not able to find any loco-regional recurrence in the segmentectomy group, whereas the incidence in the lobectomy group was 7.9%.

Nevertheless, the low incidence of loco-regional recurrence rate in our segmentectomy group is interesting as this procedure was associated with a shorter distance of tumour from resection margins compared to lobectomies which theoretically may predispose to increased risk of local recurrence. A recent systematic review has in fact indicated that a threshold of 10 mm is associated with an inflection of local recurrence [9] and the recent ESTS consensus statement on technical quality of segmentectomy recommended this distance as the minimum safe value to achieve when planning and performing a segmentectomy [10].

In our study, the proportion of patients with a tumour–margin distance shorter than 10 mm was nearly four-fold higher in the segmentectomy group compared to the lobectomy. Some other studies in the past have shown that a close resection margin was not necessarily associated with increased local recurrence [11, 12]. Interestingly, the proportion of patients with microscopically positive resection margins or uncertain margins was similar in both groups and there was no statistical difference in loco-regional recurrence rate between procedures in this high-risk subset of patients. On the other hand, the loco-regional recurrence rate was much higher in the lobectomy patients without compromised resection margins (pR0) compared to their segmentectomy counterparts (8.4% vs.1.8%, *P* = 0.033).

One finding warranting discussion is the higher rate of nodal upstaging in the lobectomy group (13% vs 3.4%). We must stress the fact that this is not a randomized trial and patients were selected to undergo either lobectomy or segmentectomy. Despite we did not include in this comparison patients with tumours located in the inner two-third of the lung or with a radiologic size of >2 cm, there may still have been tumour-related factors which played a role in deciding to perform a lobectomy. In fact, despite matching, tumours in the lobectomy group were slightly larger (median size 19 vs 14 mm) and were more frequently solid (56% vs 32%) and hypermetabolic (86% vs 54%), all characteristics which indicate a biologically more aggressive nature and may have pushed the surgeon to perform a lobectomy instead of a segmentectomy. These features may also be the main reason to explain a higher incidence of nodal upstaging in the lobectomy group.

Other retrospective studies had shown that in well-selected patients with clinical stage IA tumours smaller than 2 cm segmentectomy compares favourably with lobectomy. A recent meta-analysis, including 14 studies (12 from Asia and 2 from North America), was not able to find any difference in OS, recurrence-free survival or cancer-specific survival between segmentectomies and lobectomies in this specific group of patients [13]. Notably, there was no study published from European centres.

Stamatis *et al.* [14]. recently published the only European randomized trial comparing lobectomies and segmentectomies for patients with clinical stage IA NSCLC smaller than 2 cm. In a small population of 108 patients (54 per group), they were not able to find any difference in OS and DFS between the 2 procedures. Nodal upstaging was 7.5% after segmentectomy and 5.5% after lobectomy. Similarly, the loco-regional recurrence rate was not different between lobectomies and segmentectomies (7.4% vs 5.7%). The main difference of this study compared to the present analysis is the fact that the majority of patients were operated through an open approach.

A more recent metanalysis including 10 comparative studies (only 1 from Europe) of patients undergoing segmentectomies or lobectomies for pathologic stage I NSCLC through a minimally invasive approach was not able to find any difference in OS and DFS between the 2 procedures [15]. The only retrospective study from Europe included in the above-pooled analysis [16] compared in an unmatched fashion 96 consecutive VATS segmentectomies and 92 VATS lobectomies for clinical stage IA tumours smaller than 2 cm. After a follow-up of about 2 years, the authors were not able to find any difference in OS between the 2 procedures with an estimated 3-year survival of 93% after segmentectomy (vs 92% after lobectomy). Notably, 8% of patients in that series had carcinoid tumours which were excluded from our study.

We think our study adds to the current evidence pool especially contributing to expand the European population, so far very scant. Our findings appear to support the fact that segmentectomy is at least equivalent to lobectomy in well-selected patients with clinically stage IA NSCLC smaller than 2 cm peripherally located.

### Limitations

The findings from this study should be interpreted taking into account some limitations.

- This is a retrospective analysis. Despite the careful selection of peripheral tumours with a radiologic size smaller than 2 cm and the use of propensity score matching, inherent selection biases still remain as shown by the fact patients in the lobectomy group had larger and more frequently solid tumours with higher PET SUVmax. This study reflects the real-world scenario where all patients with a clinical stage IA are discussed in a multidisciplinary setting and multiple factors are taken into account to decide the most appropriate treatment including the extent of resection.
- Compared to the CALGB and JCOG trials [1, 2], in which all patients were considered fit for lobectomies, in our series 20% of the analysed segmentectomies were performed in patients deemed at high risk for lobectomy. Compromise segmentectomies have been associated with poorer outcome compared to intentional ones [17].
- In addition, as discussed above, also patients with subsolid lesions or part solid tumours and consolidation-to-tumour ratio lower than 0.5 were included in this study, reflecting an ever more common situation due to the implementation of the lung cancer screening program, which in our region started in November 2018. Recent European guidelines recommend a sublobar resection to treat these types of tumours [18].
- The individual surgeon’s experience with the procedure played a role in deciding the type of resection as most of the segmentectomies were performed by a single surgeon (71%). In our unit, lung cancer patients are generally referred from the MDT to the first available slot in the surgical clinic. Surgeon allocation may have had an influence in determining the extent of surgery. This is explained by the fact that this series spans back to 2017 when the results from the phase 3 trials were still not known and some of the surgeons in our units still preferred to offer lobectomy to stage I NSCLC. We acknowledge that this may represent a bias which is difficult to control for.
- An important limitation is the asymmetrical follow-up between the 2 procedures (median 22 months for segmentectomies and 49 months for lobectomies), which is explained by the fact more and more segmentectomies have been performed in the most recent period due to the ever-increasing experience and confidence with the technique and a general adoption within the team. The rate of segmentectomies among peripheral tumours with a clinical stage IA NSCLC during the first 2 years of the study time (2017 and 2018) was 18% and increased to 54% during the last 2 years (2021 and 2022). The current findings will need further verification at subsequent follow-up times.
- Notably, we only recently have started to use 3D model reconstruction to precisely localize the tumour and identify the virtual margin. None of the patients included in this analysis had a 3D CT reconstruction as part of the preoperative work-up.
- Finally, it is not our policy to systematically perform frozen section examination of the segmental or hilar nodes unless there is a strong suspicion during operation. In case of unexpected positive N1 or N2 disease at the definitive pathologic report, these patients are offered adjuvant systemic treatment in our setting.

## CONCLUSIONS AND IMPLICATIONS

Taking into account the above-mentioned limitations, we found that in a series including patients operated in a European unit and with peripheral small NSCLC clinically staged IA1–2, segmentectomy was associated with a similar outcome compared to lobectomy. We strongly encourage units to perform similar independent audits of their practice when deciding to implement this program to verify whether their indications are appropriate. In 2019, our centre commenced a regional lung cancer screening program which has progressively led to an increase in surgical referral of patients with clinical stage T1aN0. The results of the present analysis have been vital in this regard as they were used to inform a collegial multidisciplinary discussion to support a parenchymal sparing approach for screen-detected small lung cancers agreed by all components of the tumour Board. In addition, our results are currently used when discussing the pros and cons of this operation with patients in the clinic. We feel that along with existing published evidence, data reflecting the local practice are equally important to provide the patients a fair and balanced information.

Data are being continuously collected and periodically checked to monitor the outcome as a part of an ongoing quality improvement initiative.

## Data Availability

The data underlying this article will be shared on reasonable request to the corresponding author.

## Notes

Presented at the 31st European Conference on General Thoracic Surgery, Milan, Italy, 4–6 June 2023.

## ABBREVIATIONS

DFS: Disease-free survival
EFS: Event-free survival
NSCLC: Non-small-cell lung cancer
OS: Overall survival
